# Waist-to-height ratio identifies children with lower physical activity and reduced cardiorespiratory fitness: Longitudinal evidence from Norwegian primary schools – The Health Oriented Pedagogical Project (HOPP)

**DOI:** 10.1371/journal.pone.0351792

**Published:** 2026-06-18

**Authors:** Per Morten Fredriksen, Nandu Goswami, Asgeir Mamen

**Affiliations:** 1 Department of Public Health and Sport Sciences, Faculty of Social and Health Sciences, Section for Public Health, University of Inland Norway, Elverum, Norway‌‌; 2 Center for Space and Aviation Medicine, College of Medicine, Mohammed Bin Rashid University of Medicine and Health Sciences, Dubai, United Arab Emirates‌‌; 3 Gravitational Physiology and Medicine Research Unit, Division of Physiology and Pathophysiology, Otto Loewi Research Center, Medical University of Graz, Graz, Austria; 4 Kristiania University College, School of Health Sciences, Oslo, Norway; Faculty of Physical Education and Sports at Pedagogical University of Maputo, MOZAMBIQUE

## Abstract

**Objectives:**

Childhood obesity and physical inactivity are major global health concerns because of their links to cardiometabolic risk factors that may persist into adulthood. Waist-to-height ratio (WHtR) has emerged as a practical indicator of central adiposity and metabolic risk. Moderate-to-vigorous physical activity (MVPA) and cardiorespiratory fitness are important factors in reducing obesity-related risk. This study investigated the association between WHtR, physical activity, and fitness in children aged 6–12 years.

**Methods:**

The Health Oriented Pedagogical Project (HOPP) is a longitudinal cohort study conducted from 2015 to 2020 and including 2297 Norwegian children. WHtR was examined as the main time-varying exposure. Separate linear mixed models were fitted for MVPA, sedentary time, Andersen intermittent running test performance, and VO₂peak as outcomes, with age, sex, and socioeconomic status included as covariates.

**Results:**

Each 0.1 unit increase in WHtR was linked to a −0.62 min/day reduction in average MVPA. Higher WHtR was associated with lower MVPA and lower cardiorespiratory fitness, including both Andersen test performance and VO₂peak. No clear association was observed between WHtR and sedentary time.

**Conclusion:**

In this longitudinal cohort, higher WHtR was associated with lower physical activity and lower cardiorespiratory fitness. WHtR may therefore serve as a simple screening indicator for identifying children who may warrant closer assessment of fitness, physical activity, and cardiometabolic risk.

## Introduction

Childhood obesity and inactivity are growing global health concerns due to their strong links to cardiometabolic risk factors persisting into adulthood [1]. Body Mass Index (BMI), is widely used despite its limitations, does not distinguish between fat and muscle mass, nor account for fat distribution or differences across age, sex, and ethnicity. Waist-to-height ratio (WHtR), on the other hand, does offer an estimate of central adiposity and total body adiposity [2]. WHtR was therefore selected as the primary anthropometric indicator due to its incorporation of abdominal fat, all the while while remaining simple to measure in routine clinical settings. Compared with BMI, WHtR more directly reflects central adiposity, which is more closely linked to cardiometabolic risk, and it can often be interpreted using a common threshold across school-age children [2–5]. Recent pediatric work has further strengthened the clinical relevance of WHtR by showing improved alignment with adiposity- and obesity-related risk compared with BMI, and by linking WHtR-based classifications to outcomes relevant to hypertension, liver steatosis, and metabolic dysfunction [6–10]. These developments make it increasingly important to understand whether higher WHtR in childhood is also associated with lower physical activity and poorer fitness, two modifiable factors of future cardiometabolic health [2–5]. WHtR ≥ 0.5 is a widely used threshold for overweight, central adiposity, and total body adiposity and is strongly linked to cardiovascular disease risk [11].

Moderate-to-vigorous physical activity (MVPA) is central to reducing obesity-related health risk in children [12]. Higher MVPA levels are associated with healthier body composition, whereas sedentary behavior is linked to greater fat mass and lower aerobic fitness [13–15]. Sedentary behavior is a distinct behavioral domain with physiological consequences that are not fully captured by MVPA alone, which supports examining sedentary time separately [15]. Cardiorespiratory fitness, commonly assessed by VO₂peak, is a robust indicator of cardiovascular health and is associated with lower mortality and reduced risk of chronic disease [16,17]. However, direct assessment of aerobic capacity is both time-consuming and resource-intensive. Field-based tests such as the Andersen intermittent running test therefore provide a practical alternative for evaluating fitness in larger groups of children simultaneously [18]. Given the growing interest in WHtR as a practical pediatric marker of adiposity and cardiometabolic risk, it is important to examine whether higher WHtR is associated with lower objectively measured physical activity and poorer cardiorespiratory fitness in childhood. Demonstrating such associations would further support the clinical and school-health relevance of WHtR as an early screening indicator. We hypothesized that higher WHtR would be associated with lower MVPA and lower cardiorespiratory fitness, whereas the association with sedentary time would be weaker.

## Methods

The Health Oriented Pedagogical Project (HOPP), established in 2015, was designed as a prospective school-based cohort study in which pupils in all elementary schools in Horten municipality, Norway, were offered 45 minutes of additional physical activity integrated into the curriculum each school day. The sampling framework was pragmatic rather than random: seven elementary schools in Horten municipality constituted the intervention arm, whereas two schools from the greater Oslo area served as controls. As school allocation was not randomized, the study should be regarded as a controlled longitudinal cohort study rather than a randomized trial. Detailed descriptions of the recruitment process and intervention have been published previously [19].

### Study sample

The baseline cohort comprised 2297 (82%) children, n = 1542 in intervention schools and n = 752 in control schools. Repeated measurements were obtained annually from 2015 to 2020; the number of observations declined over time as older pupils transitioned from primary to secondary school, and the planned 2021 follow-up was cancelled because of the COVID-19 pandemic. Sample size also varied by variable and year because not all children completed all assessments at each wave. The largest samples are available for WHtR, MVPA, sedentary time, and Andersen test performance, whereas VO₂peak was available only in a substantially smaller subsample each year as only children born 2008 (the youngest) was included (Table 1). Missing data were handled by maximum likelihood estimation within the mixed-model framework under the missing-at-random assumption; no imputation was performed.

**Table 1:** Number of participants with available data for each study variable by test year.

	2015	2016	2017	2018	2019	2020
**WHtR**	2179	2113	1625	1163	823	518
**MVPA**	2123	1888	1452	1062	790	322
**Sedentary**	2123	1888	1452	1062	790	322
**Andersen test**	2010	1951	1513	1113	763	499
**VO**_**2**_ **peak**	189	84	188	191	199	173

The cohort was drawn from Norwegian primary schools in two geographically different areas. Socioeconomic status (SES), based on parental education level, was registered at study start in 2015, with secondary school (N = 53), high school (N = 512), bachelor degree (N = 653) or master degree/PhD (N = 473) as the highest educational level. The control schools were located in areas with somewhat higher SES than the intervention schools, which should be considered when interpreting between-school comparability and external generalizability.

### Anthropometric measures

All participants were tested during school hours at baseline and annually between 2015 and 2020 using standarized procedures. Body height (cm) was measured without shoes using a SECA 213 stadiometer (SECA GmbH, Hamburg, Germany) to the nearest 0.5 cm. Body mass (kg) was measured barefoot, in light clothing, using a Tanita MC-980MA BIA electronic scale (Tanita Corporation, Tokyo, Japan). To compensate for clothes, 0.4 kg was subtracted from the total weight. WC was measured to the nearest 0.5 cm with an anthropometric non-elastic measuring tape after normal expiration at the level of the umbilicus [20]. All variables were measured once each year, and WHtR was calculated by dividing WC by height.

### Fitness

Endurance was measured using the Andersen intermittent running test [18]. The Andersen test was conducted as a field-based intermittent endurance test in the school gymnasium. The children ran at intervals of 15s and 15s of rest for 10 minutes. Total distance equaled the number of laps completed, and measured in meters [18].

Direct measuring VO_2peak_ was limited to 1^st^-grade pupils in 2015. The same pupils were tested annually until HOPP was terminated in 2020. An incremental treadmill protocol started at 5 km·h^-1^ with a 5% incline for 5 minutes. The speed was further increased to 7 km·h^-1^ with an increase of 1 km·h^-1^ every minute until 10 km·h^-1^. After this, the inclination increased by 1% every minute until exhaustion [21]. In 2015, breath-by-breath analysis was averaged across 30-s intervals with a K4b^2^ (Cosmed Sri, Rome, Italy). From 2016 onwards, the successor, the K5 metabolic analyser with mixing chamber measurements, was used. HR during the test, the highest registered HR (HRpeak), and the time until exhaustion (TTE) were documented separately.

### Physical activity

ActiGraph wGT3X-BT accelerometers (ActiGraph LLC, Pensacola, FL, USA) were used in measuring PA. Children were instructed to attach the ActiGraph on the right hip with an elastic band and wear it at all hours unless injured, ill, absent, showering, or swimming, for seven consecutive days. Sampling frequency was 100 Hz at 10 s epochs. A minimum of 8 h/day of registered activity was required for data analysis. Non-wear time was removed using the Troiano technique with 60 minutes of consecutive zeroes and 2 minutes of activity tolerance. Valid hours were defined as 06:00–23:59. Categorical division of PA levels was based on mean counts per minute (cpm) as sedentary (0–99 cpm), light (100–1999 cpm), moderate (2000–4999 cpm), and vigorous (≥5000 cpm), recording minutes in each intensity domain (Troiano et al. 2008). Moderate to vigorous physical activity (MVPA) was calculated by summing minutes in moderate and vigorous intensity domains.

### Analyses

HOPP’s primary objective was to enhance physical activity and academic performance rather than to influence WHtR. Descriptive analysis showed minimal clinically relevant changes in WHtR and a significant difference in WHtR was observed between intervention and control schools, both consistently over time. The Shapiro-Wilk test confirmed normality of continuous variables. The health-related outcomes of physical activity levels (MVPA, sedentary behavior), and fitness measures (VO_2peak_, Andersen intermittent running test) were analyzed according to WHtR cut-off point of 0.5.

A total of 8421 measurements were recorded, with 84% (n = 7075) having a WHtR below 0.5 and 16% (n = 1346) above 0.5, consistent with national reference values for overweight using isoBMI in Norwegian children [22]. Linear mixed models (LMM) to account for the repeated measures structure of the data was used. The LMM accounted for within-subject correlation by including a random intercept for each participant. Annual measurements from 2015 to 2020 defined the repeated-measures structure, and age was entered as a continuous covariate to capture developmental change both between and within participants. Models were estimated using restricted maximum likelihood (REML) and applied a diagonal covariance structure for the repeated measures. Least squares means (adjusted means) were computed at the sample mean of the covariates.

Separate linear mixed-effects models were fitted for MVPA, sedentary time, Andersen test distance, and VO₂peak. Repeated measurements were nested within children, and participant ID was included as a random intercept to account for within-child correlation across years. WHtR was entered as the main exposure of interest, and age and sex were entered as fixed effects. Sensitivity analyses adjusted for intervention/control group to address contextual differences between participating schools The relative association between WHtR and health risk indicators in children was evaluated. β represents the regression coefficient from the linear mixed model, while Cohen’s d is presented as a standardized measure of effect size.

All regression analyses were performed using NCSS 2024 (v24.0.1). Other statistical analyses were done on Statistical Package for the Social Sciences (SPSS) version 28 (IBM, Armonk, NY, USA), with significance set at α = 0.05. The results are presented with corresponding 95% confidence intervals and p-values.

### Ethics and consent

The procedures and methods used in the study adhere to the ethical guidelines defined by the World Medical Association’s Declaration of Helsinki. The Regional Committee for Medical Research Ethics (REK) has approved the research protocol (ref.no.: 2014/2064/REK). The research is catalogued in Clinical Trials (ClinicalTrials.gov Identifier: NCT02495714) and registered 20. June 2015. All identifiable data collected were replaced by a unique identification code and were transferred digitally to a secure database during annual testing. Thus, during analysis, all identifiable personal data were deleted. Parents and legal guardians of all participants have provided informed consent.

## Results

### Anthropometric data

The descriptive statistics in Table 2 summarize weight, height, and WHtR for boys and girls from 2015 to 2020, and show an expected and consistent increase in both weight and height across the study period. Earlier publications from HOPP provide an overview of the participants’ anthropometric characteristics [22,23]. WHtR remains stable over time for both sexes, though boys generally have higher values for weight and height, WHtR differences are minimal.

**Table 2:** Descriptive statistics for age, weight, height, moderate-to-vigorous physical activity, sedentary behavior, peak oxygen uptake and weight-to-height ratio from 2015 (baseline) to 2020.

							Boys							Girls		
		**Age**	**Weight** **(kg)**		**Height** **(cm)**	**MVPA**	**SED**	**VO**_**2**_ **peak**	**WHtR**	**Age**	**Weight** **(kg)**	**Height** **(cm)**	**MVPA**	**SED**	**VO**_**2**_ **peak**	**WHtR**
**N**	**2015**	1150	1150		1150	1069	1069	118	1099	1122	1121	1122	1054	1054	100	1080
**Mean**		9,4	33,4		138,7	95,7	3482	33,4	0,45	9,4	33,3	138,3	85,6	3540	32,3	0,45
**SD**		1,77	9,22		11,49	28,55	765	4,28	0,04	1,76	9,90	12,05	26,8	729	5,27	0,05
**N**	**2016**	1085	1075		1085	943	943	43	1071	1049	1041	1049	944	944	40	1042
**Mean**		10,4	38,4		144,5	94,2	3057	31,6	0,45	10,4	38,2	144,0	76,9	3299	30,9	0,44
**SD**		1,75	10,56		11,64	31,9	972	4,07	0,05	1,74	11,00	12,17	24,66	882	4,11	0,05
**N**	**2017**	837	823		837	697	697	99	829	810	785	810	726	726	90	795
**Mean**		10,7	39,1		146,4	90,2	2829	32,8	0,45	10,8	39,2	146,2	75,1	3123	29,5	0,45
**SD**		1,43	10,28		10,38	32,7	1073	5,82	0,05	1,43	10,58	10,71	24,4	891	8,22	0,05
**N**	**2018**	613	609		613	521	521	101	592	590	586	590	516	516	80	571
**Mean**		11,1	41,2		149,0	88,0	3019	28,7	0,46	11,2	41,4	149,0	71,1	3201	27,9	0,45
**SD**		1,16	10,28		9,36	28,9	1131	5,62	0,05	1,15	10,72	9,74	24,9	981	6,09	0,05
**N**	**2019**	445	445		445	387	387	103	434	402	401	402	377	377	90	388
**Mean**		11,7	43,3		152,4	77,5	3029	26,8	0,45	11,7	43,6	152,3	68,9	3206	26,2	0,45
**SD**		0,89	10,17		9,18	27,3	1161	5,73	0,05	0,86	9,82	8,53	22,76	1048	3,80	0,05
**n**	**2020**	282	282		281	163	163	96	271	261	261	258	158	158	77	247
**Mean**		12,2	45,8		154,8	79,1	2873	23,9	0,45	12,1	46,6	154,9	67,0	3031	22,9	0,44
**SD**		0,57	10,58		8,52	30,00	1135	3,43	0,05	0,58	10,56	8,17	21,17	1069	3,22	0,06

Age (Years), Waist-to-height ratio (WHtR), Moderate-to-vigorous physical activity (MVPA, min/day), Sedentary behavior (min/day), Peak oxygen uptake (VO_2_peak, ml kg^-1^ min^-1^), Standard Deviation (SD), N = number of measurements.

### Average MVPA

**Fig 1:**
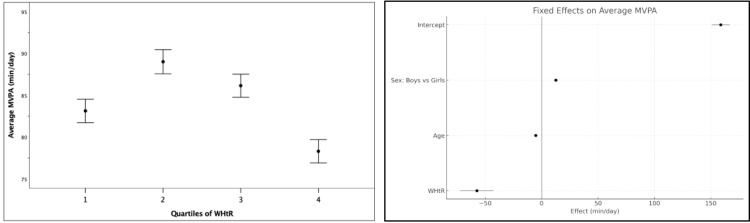
The average MVPA (min/day) across quartiles of WHtR with 95% confidence intervals (left). The fixed effect estimates for average MVPA from the linear mixed model, including sex (boys vs girls), age, and WHtR, with 95% confidence intervals are displayed (right).

Average MVPA varied across WHtR quartiles, with the highest mean level observed in the second quartile and lowest in the fourth quartile (Fig 1), hence, children in the highest WHtR quartile accumulated fewer minutes of MVPA per day than those in the lower quartiles. The adjusted mean MVPA across years and covariates was 83.7 minutes/day (95% CI: 82.8–84.7). Fig 1 illustrates that boys have higher levels of MVPA compared to girls (β = 12.61, p < 0.001). Increasing age is associated with lower MVPA (β = −5.23, p < 0.001), and higher WHtR is negatively associated with MVPA (β = −57.40, p < 0.001). Sex (d = −0.222) and age (d = −0.271) both showed a small effect size. SES (d = 0.093) and WHtR (d = −0.101) displayed very small effect.

### Average sedentary activity

**Fig 2:**
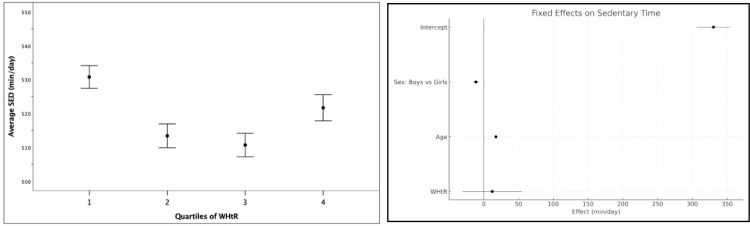
The average sedentary behavior (min/day) across quartiles of WHtR with 95% confidence intervals (left). The fixed effect estimates for sedentary behavior from the linear mixed model, including sex (boys vs girls), age, and WHtR, with 95% confidence intervals are displayed (right).

Sedentary time showed only modest variation across WHtR quartiles (Fig 2). The lowest mean sedentary time was seen in the middle quartiles, whereas somewhat higher values were observed in the first and fourth quartiles. The adjusted mean sedentary time across years and covariates was 511.9 minutes/day (95% CI: 509.70–514.20). Boys had significantly lower sedentary time than girls (β = −11.46, *p* < 0.001). Sedentary time increased with age (β = 17.42, *p* < 0.001). No significant association was found between sedentary time and WHtR (β = 12.09, *p* = 0.58). For sedentary behavior, the effect sizes were consistently very small, sex (d = 0.090), age (d = 0.078), SES (d = −0.023), and WHtR (d = 0.011).

### Andersen intermittent running test

**Fig 3:**
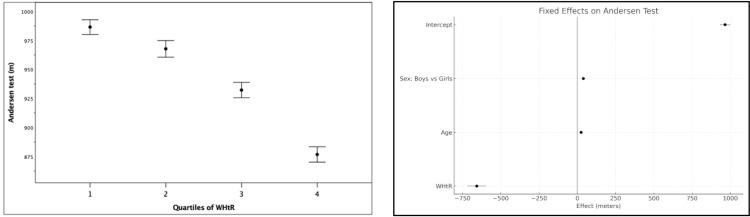
The Andersen Intermittent Running test (meters) across quartiles of WHtR with 95% confidence intervals (left). The fixed effect estimates from the linear mixed model, including sex (boys vs girls), age, and WHtR, with 95% confidence intervals are displayed (right).

Andersen test performance declined progressively across increasing WHtR quartiles (Fig 3) with children in the WHtR Q1 achieved the longest running distance, while those in Q4 had the shortest. The adjusted mean test distance was 946.4 meters (95% CI: 942.6–950.2). Boys performed significantly better than girls (β = 38.96, p < 0.001), and performance increased with age (β = 24.61, p < 0.001). Higher WHtR was strongly associated with lower performance (β = −657.33, p < 0.001). Age (d = 0.404) revealed a moderate effect size, while sex (d = −0.168) and SES (d = 0.160) had small effects. WHtR showed a small negative effect (d = −0.273), suggesting that performance increased primarily with age, whereas higher WHtR was associated with poorer test performance.

### Oxygen uptake

**Fig 4:**
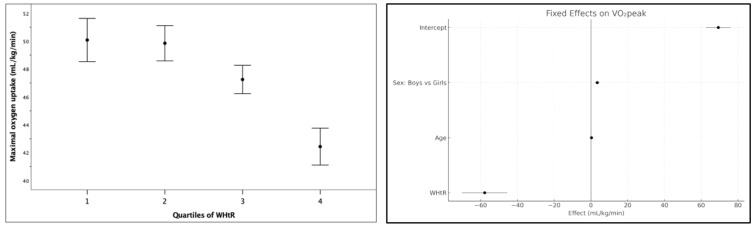
The VO_2_peak (mL·kg ⁻ ¹·min ⁻ ¹) across quartiles of WHtR with 95% confidence intervals (left). The fixed effect estimates from the linear mixed model, including sex (boys vs girls), age, and WHtR, with 95% confidence intervals are displayed (right).

VO_2_peak showed an inverse pattern across WHtR quartiles, with the highest values in the first two quartiles and progressively lower values in the third and fourth quartiles (Fig 4). The adjusted overall intercept for VO₂peak was 69.29 mL·kg ⁻ ¹·min ⁻ ¹ (95% CI: 62.53–76.04). Boys demonstrate a 3.41 ml·kg ⁻ ¹·min ⁻ ¹ higher VO₂peak than girls (p < 0.001). WHtR was negatively associated with VO₂peak (β = −57.87, p < 0.001) and age showed a non-significant positive trend (β = 0.27, p = 0.09), suggesting minimal age-related increases in VO₂peak within the studied age span. WHtR (d = −0.334) displayed a small to moderate negative effect. Sex (d = −0.152), age (d = 0.051) and SES (d = −0.015) showed small effects.

Each 0.1 unit increase in WHtR was linked to a −0.62 min/day reduction in average MVPA, 7.80 meters shorter run in the Andersen test, and −0.67 ml·kg^-1^·min^-1^ lower VO_2_peak, with no association with sedentary behavior (Table 3). Sex differences revealed that girls on average had 13 min less average MVPA per day, ran 46 meters shorter, 2.8 ml·kg^-1^·min^-1^ lower VO_2_peak, and was 164 min more sedentary time daily than boys. Age was associated with a 4.6 min/day decline in average MVPA, older children ran 32 meters farther per year and sedentary behavior rose by 41 min/day. VO_2_peak increased by 0.28 ml·kg^-1^·min^-1^ per year though were not significant. Every increase in education level in parents, as a proxy for SES, showed an increase in average MVPA of 3 minutes/day and 16 meters longer run in the Andersen test. No association was found for sedentary behavior or VO_2_peak.

**Table 3:** Associations of sex, age, SES, and WHtR with average MVPA, sedentary behavior, peak oxygen uptake, and Andersen test performance.

	Average MVPA			Sedentary behavior			Peak oxygen uptake			Andersen test		
	β	α	*d*	β	α	*d*	β	α	*d*	β	α	*d*
**Sex**	−12.99	<.001	−.222	164.4	<.001	.090	−2.79	<.001	−.152	−45.96	<.001	−.168
**Age**	−4.61	<.001	−.271	41.39	<.001	.078	.279	.164	.051	32.67	<.001	.404
**SES**	3.22	<.001	.093	−25.07	.103	−.023	−.156	.685	−.015	25.80	<.001	.160
**WHtR**	−0.62	<.001	−.101	2.12	.438	.011	−.668	<.001	−.334	−7.80	<.001	−.273

Waist-to-height ratio (WHtR), average moderate-to-vigorous-physical-activity (average MVPA), peak oxygen uptake (VO_2_peak, ml kg^-1^ min^-1^), Andersen intermittent running test (Andersen test), socioeconomic status (SES), Regression coefficient (β), p-value (α), Effect size (Cohen’s *d*).

Overall, higher WHtR was consistently associated with less MVPA and lower cardiorespiratory fitness. The magnitude of these associations was clinically meaningful, whereas sedentary time showed little or no association with WHtR.

## Discussion

In this longitudinal cohort of Norwegian primary school children, higher WHtR was consistently associated with lower MVPA and poorer cardiorespiratory fitness, whereas the association with sedentary time was weak. These findings support WHtR as a simple anthropometric indicator that may help identify children with less favorable physical activity and fitness profiles.

### Age and sex

The present analysis showed small but significant associations of both age and sex with WHtR, with a slight increase in WHtR across age in this cohort. This is plausible as children approach puberty and body composition begins to change. However, earlier HOPP data from a subsample of 6–10-year-olds showed that WHtR remained relatively stable over four years, with divergence first appearing around 12 years of age, suggesting pubertal influences [16]. This relative stability also supports an important advantage of WHtR over BMI, as WHtR appears less affected by age- and sex-related variation during growth [4,24]. Sex differences were also significant, although small in absolute terms. This is in line with a large Chinese study of 7–18-year-olds reporting marginally higher WHtR in boys than girls [25]. Overall, sex differences in WHtR during mid-childhood appear limited, supporting the view that WHtR is relatively robust across childhood.

Boys had higher physical activity and fitness than girls, consistent with previous studies [26,27]. Girls accumulated less daily MVPA, ran shorter distances, had lower VO_2_peak, and spent more time sedentary, likely reflecting both biological and social influences [28]. Age was negatively associated with MVPA, declining by about 5 min/day per year, in line with previous research [29], whereas fitness improved, likely due to growth-related gains in leg length, biomechanics, and muscle mass [30]. Although small age- and sex-related differences in WHtR were observed, these do not undermine its usefulness. Evidence suggests that WHtR does not require age- and sex-specific percentiles between approximately 6 and 18 years [31]. Taken together, the findings support WHtR as a practical marker across childhood, while also underscoring the importance of maintaining physical activity and fitness as children grow older.

### Aerobic power and fitness

The LMM results showed a significant negative association between WHtR and both VO_2_peak and fitness performance, indicating that higher WHtR was associated with lower aerobic power and poorer running performance. This is consistent with previous studies showing that higher VO_2_peak is linked to healthier body composition and metabolic profiles in children [32–34], and that higher cardiorespiratory fitness is inversely associated with adiposity in youth [35]. These findings reinforce the importance of maintaining a healthy body composition to reduce future cardiovascular risk [36,37]. Similar associations have been reported in Latin American adolescents, where higher relative VO_2_peak was associated with lower BMI, waist circumference, and WHtR [35], and in South African children, where WHtR was independently negatively associated with VO_2_peak after adjustment for other risk factors [38].

Running tests provide a practical and cost-effective alternative for large-scale studies [18], and in the present study, higher WHtR was also associated with lower fitness test performance [39]. Each unit increase in WHtR was associated with 7.33 meters shorter running distance. Previous HOPP data likewise showed that WHtR was strongly associated with children’s fitness levels [22]. Together, these findings support the close relationship between central adiposity and aerobic fitness, where low fitness and high WHtR often coexist as part of a broader cardiometabolic risk profile [38]. Some evidence also suggests a non-linear association, with both very high and very low WHtR linked to lower VO_2_peak [25].

### Physical activity and sedentary behavior

Physical inactivity is a major global risk factor for non-communicable diseases and mortality, whereas regular physical activity may reduce obesity, inflammation, and insulin resistance [1,12,14,40,41]. In the present study, WHtR was negatively associated with MVPA, with the LMM indicating that each unit increase in WHtR was associated with 0.46 min/day less MVPA. This supports the view that greater physical activity contributes to a healthier body composition, in line with studies showing that regular exercise reduces excess weight gain and fat accumulation in children [42,43]. Our findings further suggest that higher MVPA and better fitness were inversely associated with WHtR after adjustment for key confounders, adding to previous evidence through the use of detailed MVPA and fitness assessments [3,44,45].

Several mechanisms may explain these associations. Greater central adiposity may increase the mechanical cost of movement, impair running economy, and be linked to metabolic and inflammatory profiles that reduce exercise tolerance. Behavioral factors may also contribute, as children with higher adiposity may participate less in vigorous play and endurance activities, reinforcing a cycle of lower activity and fitness. These findings support inclusive school-based physical activity strategies that promote regular, enjoyable, and non-stigmatizing opportunities for MVPA.

Although sedentary behavior has previously been linked to obesity [1,46], no association was found between WHtR and sedentary time in the present study. This may reflect uniformly high sedentary time across WHtR groups or limitations in accelerometer-based assessment of some sedentary behaviors. Girls were more sedentary, and sedentary time increased slightly with age, but sedentary behavior alone did not explain the differences observed in physical activity.

### Cardiometabolic risk in children

The present findings are consistent with previous pediatric studies showing that WHtR is more closely related to central adiposity and cardiometabolic risk than BMI alone, and with studies linking greater adiposity to lower cardiorespiratory fitness [2–5,25,47]. The current study extends this literature by demonstrating similar patterns in a longitudinal school-based cohort with repeated objective measures of physical activity and repeated assessments of fitness.

WHtR is increasingly recognized as a simple and clinically meaningful marker of central adiposity in children [2,4,48,49]. Unlike BMI percentiles, it reflects abdominal fat distribution more directly and has the practical advantage, in most populations, of being usable across age and sex groups in school-aged children with a common cutoff [31]. A WHtR threshold of 0.50 is widely recommended to indicate increased central adiposity and elevated cardiometabolic risk [31,49,50]. International data from more than 24,000 children across 10 countries support a cutoff near 0.5 in most populations, although some ethnic variation has been reported [2,47]. Agbaje et al. further proposed age- and sex-specific pediatric WHtR thresholds for high and excess fat mass, derived against DXA, with good classification accuracy and clear clinical relevance [5].

Previous work has also shown that BMI may overestimate overweight prevalence and miss children with elevated central adiposity identified by WHtR [4,5]. In addition, several adiposity indices have been associated with aerobic performance in children, explaining a substantial proportion of the variance in fitness [51]. Earlier HOPP results likewise showed that WHtR was a better indicator of fitness than BMI and waist circumference [52]. In the present study, the association between higher WHtR and lower VO_2_peak further supports the view that central adiposity and aerobic fitness are closely linked.‌‌

In practice, WHtR can be obtained quickly from routine height and waist measurements in school health services or pediatric primary care. Used alongside clinical judgment, it may help identify children who would benefit from further assessment of physical activity, aerobic fitness, and broader cardiometabolic risk [5,6,9,53]. The present findings therefore place higher WHtR, lower physical activity, and lower aerobic fitness within the same broader pattern of unfavorable health risk already evident in childhood.

### Limitations

Because the participating schools were not randomly selected and differed in geographic and socioeconomic context, the findings may not generalize directly to more socioeconomically diverse or ethnically heterogeneous pediatric populations. The homogeneity in body composition in the present sample may limit generalizability, especially to populations with wider WHtR variation. Also, the observational cohort design supports longitudinal associations but does not establish causality. In addition, accelerometers provide objective activity data but may underestimate cycling, swimming, upper-body activity, and some context-specific sedentary behaviors.

## Conclusion

The HOPP study demonstrates that higher WHtR in childhood is associated with lower physical activity, poorer aerobic fitness, and emerging age- and sex-related changes in body composition. WHtR may therefore be useful as a simple indicator for early risk stratification, but it should not be interpreted as a direct determinant of activity or fitness. These findings support WHtR as a practical and informative marker of pediatric health. Routine monitoring of WHtR, alongside efforts to improve children’s physical activity and fitness, may help identify and mitigate early cardiometabolic risk. Promoting an active lifestyle and improving aerobic fitness in primary school children may yield dual benefits by enhancing fitness and reducing central adiposity, thereby supporting healthier trajectories into adolescence. Overall, the present results reinforce the value of WHtR as both a research and clinical tool for assessing cardiometabolic health in youth and highlight the interplay between lifestyle behaviors and central adiposity in childhood.‌‌

### Trial registration

Clinical Trial.gov. Identifier: NCT02495714. Registered 20 June 2015. https://register.clinicaltrials.gov/prs/app/action/LogoutUser?uid=U0002ORK&ts=13&sid=S0005MCN&cx=pc85td
