# Supplementary material for: Waist-to-height ratio identifies children with lower physical activity and reduced cardiorespiratory fitness: Longitudinal evidence from Norwegian primary schools – The Health Oriented Pedagogical Project (HOPP)
Source: PLoS One. 2026 Jun 18;21(6):e0351792. doi: 10.1371/journal.pone.0351792 (PMC13278397; doi:10.1371/journal.pone.0351792)
Supplement: Attachment — Submitted filename: Reviewer comments and responses.docx [file pone.0351792.s001.docx]

Reviewer Comments and Responses

# Reviewer #1

## Comment #1

Overall, the manuscript addresses an important public health question by examining the longitudinal relationship between waist-to-height ratio, physical activity, and cardiorespiratory fitness in a large cohort of Norwegian children. The large sample size and longitudinal design of the HOPP study provide strong potential for meaningful insights into pediatric health risk indicators. However, several methodological and reporting aspects require clarification, particularly regarding the statistical model structure, measurement procedures, and interpretation of regression results. Strengthening these areas and moderating causal interpretations would significantly improve the scientific clarity and impact of the manuscript.

### Response

Thank you for this balanced overall assessment. We agree that the manuscript would benefit from clearer reporting of the cohort design, measurement procedures, linear mixed-model structure, and interpretation of the regression coefficients. In the revised version, the methods and results sections are strengthened.

## Comment #2

The abstract contains an important methodological inconsistency: it states that WHtR was used as the independent variable, but then lists age, sex, MVPA, VO₂peak, and Andersen intermittent running test as dependent variables, which is conceptually unclear because age and sex are typically covariates or control variables rather than dependent outcomes. This needs clarification.

### Response

Thank you. The abstract is rewritten to distinguish clearly between exposure, outcomes, and covariates.

## Comment #3

The results section of the abstract focuses on statistical significance but should better explain the practical meaning of the regression coefficients (e.g., how much fitness decreases with higher WHtR).

### Response

We agree. The abstract is revised.

## Comment #4

The conclusion should avoid implying a direct causal relationship between WHtR and lower physical activity or fitness, since the design primarily demonstrates associations within a longitudinal cohort.

### Response

We agree. The Discussion and Conclusion are moderated.

## Comment #5

The introduction provides strong background on childhood obesity, physical inactivity, and cardiometabolic risk, but it places relatively little emphasis on the specific advantages of waist-to-height ratio compared with other adiposity indicators such as BMI or waist circumference alone. A clearer justification for selecting WHtR as the primary indicator would strengthen the rationale.

### Response

Thank you. We agree, and the rationale for selecting WHtR as the primary anthropometric indicator is strengthened.

## Comment #6

Several paragraphs broadly discuss physical activity and obesity in children but do not directly connect this information to the specific research objective of examining longitudinal relationships between WHtR, MVPA, and aerobic fitness. The section would benefit from a more focused narrative leading directly to the research question.

### Response

We agree. The introduction is tightened so that each paragraph leads directly to the study aim.

## Comment #7

The final paragraph should more clearly state the specific hypotheses, particularly whether the authors expected WHtR to predict lower MVPA, lower VO₂peak, or both.

### Response

We agree. The final paragraph of the introduction is revised to state the specific hypotheses explicitly.

## Comment #8

The description of the Health Oriented Pedagogical Project (HOPP) cohort is informative, but the manuscript should more clearly explain the sampling framework and school recruitment procedures, including whether schools were randomly selected or volunteered to participate.

### Response

Thank you. We agree that the recruitment framework should be described directly in this manuscript rather than only by citation to earlier HOPP papers. We clarify that the cohort was school-based and non-random, that all elementary schools in Horten municipality participated in the intervention arm, and that two comparison schools from the greater Oslo area served as controls.

## Comment #9

Because the study spans several years (2015–2020), it is important to clarify participant retention and attrition rates, including how many children were included in repeated measurements and how missing data were handled.

### Response

We agree. The revised manuscript now includes a table showing the number of participants per year.

## Comment #10

Additional detail about socioeconomic background or school characteristics would help contextualize the population and improve the interpretation of generalizability.

### Response

We agree. A short contextual description of the participating schools and socioeconomic background is added.

## Comment #11

The measurement procedures for waist circumference and height should provide clearer methodological detail (e.g., number of measurements taken, measurement reliability, and standardization procedures).

### Response

We agree. The anthropometric methods are expanded.

## Comment #12

The manuscript should more clearly explain how physical activity (MVPA) was measured and processed, including accelerometer settings, epoch length, wear-time criteria, and cut-points used to classify MVPA.

### Response

We believe this is explained in the Methods section under “Physical activity.”

## Comment #13

The relationship between VO₂peak and the Andersen intermittent running test should be clarified, particularly whether VO₂peak was directly measured or estimated from the test performance.

### Response

We believe this is already stated in the text. The description of the VO₂peak test is provided, as well as the Andersen test. To be clear, as written in the text, VO₂peak was not estimated from the Andersen test.

## Comment #14

The manuscript states that linear mixed models (LMM) were used, which is appropriate for longitudinal data, but the description of the model structure requires clarification. Specifically, it should clearly indicate which variables were treated as fixed effects and which were included as random effects (e.g., participant or school level).

### Response

We agree. The mixed-model specification is rewritten for clarity. The revised text states that repeated observations were nested within participants, participant ID was included as a random intercept, and WHtR, age, and sex were included as fixed effects. Because the number of schools was small, school is best addressed as a sensitivity covariate or fixed effect rather than as a higher-level random effect.

## Comment #15

The analysis section should clarify whether time was included as a repeated factor in the model, given the longitudinal design of the HOPP dataset.

### Response

We agree, and the text is changed to better describe the analyses.

## Comment #16

The manuscript should also explain how potential confounding variables such as age, sex, and growth-related changes were incorporated into the models.

### Response

This is now explained.

## Comment #17

The results section clearly reports associations between WHtR and fitness-related variables, but the presentation would benefit from clearer interpretation of regression coefficients, explaining their practical meaning for child health and performance.

### Response

We agree, and relevant changes have been made in the Results section.

## Comment #18

Given the large sample size (n ≈ 2297), the manuscript should place greater emphasis on effect size interpretation rather than relying primarily on statistical significance.

### Response

We agree. The text has now been changed to better accommodate effect size interpretation.

## Comment #19

The relationship between WHtR, MVPA, and VO₂peak could be presented more clearly by emphasizing the direction and magnitude of associations rather than focusing primarily on p-values.

### Response

We agree and revised the Results narrative accordingly.

## Comment #20

The discussion appropriately highlights the relationship between central adiposity and reduced physical fitness, but the explanation of mechanisms could be expanded to include physiological and behavioral pathways linking adiposity to reduced physical activity and aerobic capacity.

### Response

Thank you. We agree and expand the discussion of plausible mechanisms.

## Comment #21

The manuscript should more clearly compare the findings with previous research examining WHtR, BMI, and fitness in children, highlighting whether the current results confirm or extend existing evidence.

### Response

We agree. The revised Discussion compares the present findings more systematically with previous pediatric studies on WHtR, BMI, and fitness.

## Comment #22

The limitations section should more explicitly acknowledge several important issues, including measurement limitations of accelerometry, potential residual confounding, and the observational nature of the data, which limits causal interpretation.

### Response

We agree. The limitations section is now expanded substantially.

## Comment #23

The manuscript suggests that WHtR may be a useful screening tool for identifying children with lower physical activity and fitness; however, the practical implications should further explain how schools or health professionals could implement such screening in real-world settings.

### Response

We agree. The practical implications section is now made more concrete.

## Comment #24

The discussion could also address how school-based physical activity programs might target children with higher WHtR to improve both fitness and cardiometabolic health.

### Response

We agree. A short paragraph on how school-based interventions may respond to these findings without stigmatizing children is added to the Discussion.

## Comment #25

The conclusions should be slightly moderated to avoid implying that WHtR directly determines physical activity or fitness levels.

### Response

We agree, and the Conclusion is now moderated.

## Comment #26

The section should emphasize that WHtR is associated with lower MVPA and lower cardiorespiratory fitness, highlighting its potential value as an early screening indicator for cardiometabolic risk in children.

### Response

This is now included in the Conclusion.

# Reviewer #2

## Comment #1

This is an important and useful study examining an emerging measure of excess adiposity in detecting the risk of physical fitness; however, the authors submitted an embarrassing text. This reviewer provided comments with an interest in improving the study; otherwise, this is a straight rejection.

### Response

We thank the reviewer for recognizing the importance of the study and for providing detailed comments intended to improve the manuscript. We acknowledge that the submitted version contained major formatting and presentation problems, particularly in the reference list and tables, and we regret that these technical issues detracted from the scientific content. The revised version is substantially cleaned, reformatted, and rewritten for clarity.

## Comment #2

The article has no reference bibliography, the in-text citation has two types of referencing styles. This is unprofessional and sloppy. The authors should provide an explanation for such sloppiness.

### Response

We apologize for this formatting error. The problem was not intentional and appears to have resulted from a Zotero/Word field-update issue in the submitted version, where automatic reference updating was disabled before export. This prevented the bibliography and citation fields from refreshing correctly. The reference list and citation style have now been corrected and unified throughout the manuscript.

## Comment #3

Did the authors use ChatGPT to write the original draft of the article?

### Response

No. The manuscript was not written by generative AI. As already disclosed in the manuscript’s AI statement, ChatGPT was used only for limited English-language correction, after which the authors reviewed and edited the text and retained full responsibility for the content, interpretation, and final wording.

## Comment #4

The authors should include the latest longitudinal evidence and reviews in the introductory rationale. Remove all references >10 years old. The field is rapidly evolving.

### Response

We agree in part. The Introduction and Discussion are updated with more recent longitudinal evidence and recent reviews, particularly regarding movement behavior, pediatric adiposity, and WHtR-based risk classification. However, we do not believe that all references older than 10 years should automatically be removed, because some older sources are foundational method papers, such as the Andersen test, the 20 m shuttle run, and original WHtR rationale papers.

## Comment #5

For example, rather than cite (Tremblay et al. 2011) in Line #98 on relations between movement behaviour and adiposity, a stronger long-term evidence exists in children, https://doi.org/10.1038/s41467-023-43316-w

### Response

We agree and have supplemented the older citation with the more recent longitudinal pediatric evidence.

## Comment #6

A review on sedentary behaviour is also important to this work. https://doi.org/10.1152/physrev.00022.2022

### Response

We agree, and Pinto et al. is added, especially to frame why sedentary time may not be equivalent to low MVPA and why physiology- and posture-specific mechanisms matter.

## Comment #7

Remove all references for adults from the text and focus on children and adolescents.

### Response

We agree in principle. The revised manuscript focuses more consistently on pediatric evidence. Two methodological or conceptual references are retained.

## Comment #8

This statement has no meaning: “Tests like 20 m SRT and Yo-Yo can be problematic from a pedagogical view.” Please delete. https://doi.org/10.1152/ajpheart.00570.2025

### Response

We do not agree with this point in principle. Tests that exclude children during the assessment because they fail to maintain the required pace may be problematic in pediatric populations, as they can stigmatize children with lower physical capacity. One advantage of the Andersen test is that all children are able to complete the test. However, we agree that this point is not essential to the scientific argument of the manuscript, and the statement is therefore deleted.

## Comment #9

Focus on establishing the role of pediatric WHtR in detecting cardiometabolic risk, liver diseases, type 2 diabetes, and hypertension in the introductory rationale, and add these new references to both the Introduction and Discussion: https://doi.org/10.1038/s41366-025-01949-5 https://doi.org/10.1016/j.tjnut.2026.101426 https://doi.org/10.1093/obendo/wjaf002 https://doi.org/10.1210/jendso/bvaf079

### Response

Thank you. We agree that the rationale should be strengthened by linking WHtR not only to adiposity, but also to clinically relevant pediatric outcomes. The Introduction and Discussion are revised accordingly.

## Comment #10

“This study aims to test the WHtR’s ability to predict physical activity and fitness in children” is a weak justification. The authors should link the updated introduction to why this research question is important. One reason is the new approach to defining obesity, as emphasized in the new obesity guideline. Moreover, validating pediatric WHtR cutoff, as highlighted in the discussion, is a valid research question.

### Response

We agree, and the wording is now changed.

## Comment #11

Tables should be editable. The authors’ approach of copy-pasting photos or screenshots of tables is very poor and suggests a lack of diligence.

### Response

We agree and apologize. All tables are now editable.

## Comment #12

What does the two repeated column of WHtR represent in Table 1?

### Response

This appears to be a table-formatting or labeling error and is now corrected.

## Comment #13

Table 1 should present the baseline characteristics of the participants. It is shameful that there is no information.

### Response

We agree. A table presenting baseline participant characteristics is added (now Table 2).

## Comment #14

Prepare a table showing the values of WHtR per quartile in this cohort.

### Response

We agree, but rather than a table we included the results in the figures. All figures now also include all values per WHtR quartile.

## Comment #15

Reanalyse your study using a pediatric validated cutoff which you have cited as reference #50.

### Response

It is reassuring that the relevant references were available, despite the claim that they were not. The internationally most accepted and validated cutoff for WHtR is 0.5, as supported by a substantial body of literature. Furthermore, a new consensus report by Zong et al. is in preparation and also adopts the 0.5 threshold. Although minor deviations from this cutoff may occur according to age, sex, or ethnicity, the practicality and simplicity of a uniform cutoff of 0.5 are generally considered to outweigh such limitations. We therefore chose to keep the analyses as they are.

## Comment #16

What is the prevalence of high and excess fat in this population based on the Waist-to-height ratio cut points: <0.40 (low fat mass), 0.40 to <0.50 (normal fat mass), 0.50 to <0.53 (high fat mass), and ≥0.53 (excess fat mass) in males? Waist-to-height ratio cut points for females are <0.40 (low fat mass), 0.40 to <0.51 (normal fat mass), 0.51 to <0.54 (high fat mass), and ≥0.54 (excess fat mass).

### Response

We appreciate this suggestion. However, the primary aim of the present study was not to describe the prevalence of fat-mass categories in the cohort, but to investigate the association between WHtR and physical activity and fitness outcomes. For that reason, we do not consider prevalence estimates based on sex-specific WHtR cut points essential to the main research question. In the present manuscript, WHtR is used as an anthropometric indicator to examine associations with behavioral and fitness variables, rather than as a basis for clinical classification of adiposity. In addition, this matter has earlier been described in papers from the HOPP study. We therefore chose to keep the focus of the present manuscript on the association analyses.

## Comment #17

The discussion should be entirely rewritten, not with ChatGPT as it is too long and incoherent. Use the references suggested above to improve this manuscript.

### Response

The Discussion is now rewritten and tightened, including the references mentioned.

## Comment #18

Is this a longitudinal study, what was the follow-up duration, and which measures are undertaken at baseline and follow-up?

### Response

As written in the Methods, Study Sample: “Repeated measurements were obtained annually from 2015 to 2020; the number of observations declined over time as older pupils transitioned from primary to secondary school, and the planned 2021 follow-up was cancelled because of the COVID-19 pandemic.”

## Comment #19

Statistical analysis strategy must be well written for clarity and repeatability.

### Response

We believe the description is now clearer.

## Comment #20

What does this mean? ‘Automatisk oppdatering av henvisninger er deaktivert. For å se bibliografien, klikk Oppdater i Zotero fanen.’

### Response

This message means that automatic updating of Zotero citation fields was disabled in the local Word document before export. As a consequence, the document that was submitted did not refresh the bibliography and some citation fields correctly. This was a technical formatting error, not a scientific one, and it has now been corrected.
