# Supplementary material for: Waist-to-height ratio identifies children with lower physical activity and reduced cardiorespiratory fitness: Longitudinal evidence from Norwegian primary schools – The Health Oriented Pedagogical Project (HOPP)
Source: PLoS One. 2026 Jun 18;21(6):e0351792. doi: 10.1371/journal.pone.0351792 (PMC13278397; doi:10.1371/journal.pone.0351792)
Supplement: Attachment — Submitted filename: Response 2 referees.docx [file pone.0351792.s002.docx]

**Response to reviewers**

*Title:* Waist-to-height ratio identifies children with lower physical activity and reduced cardiorespiratory fitness: longitudinal evidence from Norwegian primary schools – the Health Oriented Pedagogical Project (HOPP)

# Reviewer #1

We thank Reviewer #1 for the careful evaluation of the revised manuscript and for the positive assessment.

**Response:** We are grateful for this encouraging assessment and thank the reviewer for recognizing the revisions made to improve the manuscript.

# Reviewer #2

We thank Reviewer #2 for the positive overall assessment of the revised manuscript and for the remaining helpful comments. Each point is addressed below.

## Comment 1

**Comment:** Line 78… spelling error “therefor”

**Response:** Thank you. This has been corrected to “therefore.”

## Comment 2

**Comment:** Line 88… change “closely tied to” two modifiable factors “of” future cardiometabolic health

**Response:** Thank you. The sentence has been revised accordingly for clarity and grammatical correctness.

## Comment 3

**Comment:** Line 129… It appears that something is missing in the text “only children born 200 (the youngest)”

**Response:** Thank you. This error has been corrected in the revised manuscript.

## Comment 4

**Comment:** Line 182… typo error “HOPP’s primarily objective”… should be “primary”.

**Response:** Thank you. This has been corrected.

## Comment 5

**Comment:** Line 228… typo error “from HOPP provid an overview”…. should be “provide”.

**Response:** Thank you. This has been corrected.

## Comment 6

**Comment:** In the discussion, the authors should emphasise the significance of WHtR which was confirmed in this study regarding how WHtR overcame a major limitation of BMI variability with age and sex. The authors noted in the result section that “WHtR remains stable over time for both sexes” See the following references.

**Response:** We agree that one of the practical strengths of WHtR is that, unlike BMI, it is less dependent on age- and sex-specific interpretation across childhood. We have strengthened the discussion to emphasize this point more clearly and have incorporated the suggested references where relevant. This addition helps position WHtR more explicitly as a clinically useful marker that may overcome an important limitation of BMI in pediatric screening.

## Comment 7

**Comment:** Table 2 should include a column of mean age and SD of the boys and girls per study year and SES. Also mean (SD) for MVPA, VO2 peak and Sedentary time must be included in Table 2 per year of follow up.

**Response:** We agree that clearer descriptive information improves interpretability. We have revised the descriptive presentation accordingly in the table. The SES was registered in 2015, and the distribution is mentioned in the method section.

## Comment 8

**Comment:** There are two symbols in the result Line 243–247 that should be defined at first use (β, d).

**Response:** Thank you. We have now defined β and d in the Method section.

## Comment 9

**Comment:** This point should be included in the abstract “Each 0.1 unit increase in WHtR was linked to a -0.62 min/day reduction in average MVPA”

**Response:** We agree that the abstract should reflect the main quantitative finding more clearly. We have revised the abstract to report the WHtR–MVPA association in a clearer and more interpretable form.

## Comment 10

**Comment:** The title of Table 3 reads more like a footnote, kindly revise.

**Response:** Thank you. The title of Table 3 has been revised to make it shorter and more consistent with standard table formatting.

## Comment 11

**Comment:** The major concern is that representing WHtR in quartile is not clinically relevant. The topmost quartile equals 75th percentile. This lumps together normal WHtR fat mass, high fat mass and excess fat mass in the same category. Therefore, creating room for misclassifications. It is also impossible to state the role of excess adiposity on MVPA clearly.

Importantly, the authors response to the previous comment is not satisfactory “What is the prevalence of high and excess fat in this population based on the waist-to-height ratio cutpoints: <0.40 (low fat mass), 0.40 to <0.50 (normal fat mass), 0.50 to <0.53 (high fat mass), and ≥0.53 (excess fat mass) in males? Waist-to-height ratio cut points for females are <0.40 (low fat mass), 0.40 to <0.51 (normal fat mass), 0.51 to <0.54 (high fat mass), and ≥0.54 (excess fat mass).

This is the clinical relevance of this new study. These WHtR cutpoints represent 85th percentile to <95th percentile for high fat mass and 95th percentile and above as excess fat mass. Rather than use quartile, this validated clinical cutpoints provide detailed information that could make this study a reference in future clinical guideline update. Kindly re-analyse the data based in the provide cutpoint.

**Response:** We thank the reviewer for this thoughtful comment and for highlighting the potential clinical relevance of recently proposed WHtR cut-points. We agree that such cut-points may be useful when the purpose is to classify children into adiposity categories or to estimate the prevalence of high and excess fat mass. However, that is not the primary aim of the present manuscript.

Our study was designed to examine associations between WHtR and physical activity and fitness, not to compare predefined adiposity classes. In this context, WHtR was analysed primarily as a continuous exposure variable in the linear mixed models, which we consider the most appropriate approach for addressing the study question. The quartile presentation was included only as a descriptive and visual illustration of the graded decline in physical activity and fitness across the WHtR distribution. It was not intended as a clinical classification system.

We therefore respectfully disagree that the use of quartiles constitutes “misclassification” in the context of this manuscript. Quartiles were not used to define clinical fat-mass categories, but to display the pattern of association across the observed WHtR range. The main message is the graded relationship between increasing WHtR and lower MVPA and fitness, rather than the contrast between discrete adiposity categories.

We also examined the reviewer’s suggested WHtR cut-points in our dataset. These additional analyses did not provide materially different information from the analyses already presented. Rather, they confirmed the same overall pattern: higher WHtR was associated with higher adiposity and less favourable body-composition measures, which is fully consistent with the findings from the continuous models and quartile-based descriptive presentation. In our view, reframing the manuscript around multiple WHtR cut-points would shift the emphasis from association to classification without adding substantial interpretive value to the core message of the study.

We further note that, from a public health perspective, the WHtR threshold of 0.5 remains the most widely recognized and easily interpretable message. While more granular cut-points may be of theoretical and clinical interest, especially in body-fat classification research, their practical utility in public communication is less straightforward. For this reason, we believe that the present analytical approach better serves the aims of the manuscript.

## Comment 12

**Comment:** This statement was repeated several times in the discussion, kindly delete all redundant repetitions “these results support WHtR as a simple marker that may help identify children at risk”

**Response:** We agree that this point was stated too often in the previous version. Redundant repetitions have been removed.

## Comment 13

**Comment:** The null association between sedentary behaviour and WHtR may be due to the quartile category approach …

**Response:** We appreciate this point. However, the null finding for sedentary behavior was not based on the quartile figures, but on the linear mixed models in which WHtR was analysed as a continuous variable. The quartile presentation was descriptive only. We have clarified this more explicitly in the revised manuscript to avoid the impression that the null association was an artifact of categorization.
