# Supplementary material for: Waist-to-height ratio identifies children with lower physical activity and reduced cardiorespiratory fitness: Longitudinal evidence from Norwegian primary schools – The Health Oriented Pedagogical Project (HOPP)
Source: PLoS One. 2026 Jun 18;21(6):e0351792. doi: 10.1371/journal.pone.0351792 (PMC13278397; doi:10.1371/journal.pone.0351792)
Supplement: Peer review history

# Peer review: Decision Letter 0

**DOI:** 10.1371/journal.pone.0351792.r001

**Authors:** Timoteo Salvador Lucas Daca

29 Mar 2026

PONE-D-26-07428

Waist-to-height ratio identifies children with lower physical activity and reduced cardiorespiratory fitness: longitudinal evidence from Norwegian primary schools – the Health Oriented Pedagogical Project (HOPP).

PLOS One

Dear Dr.  Fredriksen,

Thank you for submitting your manuscript to PLOS ONE. After careful consideration, we feel that it has merit but does not fully meet PLOS ONE’s publication criteria as it currently stands. Therefore, we invite you to submit a revised version of the manuscript that addresses the points raised during the review process.

Please submit your revised manuscript by May 13 2026 11:59PM.  If you will need more time than this to complete your revisions, please reply to this message or contact the journal office at plosone@plos.org. When you're ready to submit your revision, log on to https://www.editorialmanager.com/pone/ and select the 'Submissions Needing Revision' folder to locate your manuscript file.

Please include the following items when submitting your revised manuscript:

- A letter that responds to each point raised by the academic editor and reviewer(s). You should upload this letter as a separate file labeled 'Response to Reviewers'.
- A marked-up copy of your manuscript that highlights changes made to the original version. You should upload this as a separate file labeled 'Revised Manuscript with Track Changes'.
- An unmarked version of your revised paper without tracked changes. You should upload this as a separate file labeled 'Manuscript'.

If you would like to make changes to your financial disclosure, please include your updated statement in your cover letter. Guidelines for resubmitting your figure files are available below the reviewer comments at the end of this letter.

If applicable, we recommend that you deposit your laboratory protocols in protocols.io to enhance the reproducibility of your results. Protocols.io assigns your protocol its own identifier (DOI) so that it can be cited independently in the future. For instructions see: https://journals.plos.org/plosone/s/submission-guidelines#loc-laboratory-protocols. Additionally, PLOS ONE offers an option for publishing peer-reviewed Lab Protocol articles, which describe protocols hosted on protocols.io. Read more information on sharing protocols at https://plos.org/protocols?utm_medium=editorial-email&utm_source=authorletters&utm_campaign=protocols.

As the corresponding author, your ORCID iD is verified in the submission system and will appear in the published article. PLOS supports the use of ORCID, and we encourage all coauthors to register for an ORCID iD and use it as well. Please encourage your coauthors to verify their ORCID iD within the submission system before final acceptance, as unverified ORCID iDs will not appear in the published article. *Only*   the individual author can complete the verification step; PLOS staff *cannot*   verify ORCID iDs on behalf of authors.

We look forward to receiving your revised manuscript.

Kind regards,

Timoteo Salvador Lucas Daca, Ph.D

Academic Editor

PLOS One

**Journal Requirements:**

When submitting your revision, we need you to address these additional requirements.

1. Please ensure that your manuscript meets PLOS ONE's style requirements, including those for file naming. The PLOS ONE style templates can be found at

https://journals.plos.org/plosone/s/file?id=wjVg/PLOSOne_formatting_sample_main_body.pdf and

https://journals.plos.org/plosone/s/file?id=ba62/PLOSOne_formatting_sample_title_authors_affiliations.pdf

2. We note that the grant information you provided in the ‘Funding Information’ and ‘Financial Disclosure’ sections do not match.

When you resubmit, please ensure that you provide the correct grant numbers for the awards you received for your study in the ‘Funding Information’ section.

3. Thank you for stating the following financial disclosure:

“The HOPP study is funded by Horten municipality, Kristiania

University College, the Norwegian Order of Odd Fellow Research Fund, the Oslofjord

Regional Research Fund, and the Norwegian Fund for Post-Graduate

Training in Physiotherapy.

Powered”

Please state what role the funders took in the study.  If the funders had no role, please state: "The funders had no role in study design, data collection and analysis, decision to publish, or preparation of the manuscript."

If this statement is not correct you must amend it as needed.

Please include this amended Role of Funder statement in your cover letter; we will change the online submission form on your behalf.

4. Please note that funding information should not appear in any section or other areas of your manuscript. We will only publish funding information present in the Funding Statement section of the online submission form. Please remove any funding-related text from the manuscript.

5. In the online submission form you indicate that your data is not available for proprietary reasons and have provided a contact point for accessing this data. Please note that your current contact point is a co-author on this manuscript. According to our Data Policy, the contact point must not be an author on the manuscript and must be an institutional contact, ideally not an individual. Please revise your data statement to a non-author institutional point of contact, such as a data access or ethics committee, and send this to us via return email. Please also include contact information for the third party organization, and please include the full citation of where the data can be found.

6. If the reviewer comments include a recommendation to cite specific previously published works, please review and evaluate these publications to determine whether they are relevant and should be cited. There is no requirement to cite these works unless the editor has indicated otherwise.

**Additional Editor Comments:**

-->This manuscript does not describe a technically sound piece of scientific research with data that supports the conclusions. Several methodological and reporting aspects require clarification, particularly regarding the statistical model structure, measurement procedures, and interpretation of regression results.

[Note: HTML markup is below. Please do not edit.]

Reviewers' comments:

Reviewer's Responses to Questions

**Comments to the Author**

1. Is the manuscript technically sound, and do the data support the conclusions?

The manuscript must describe a technically sound piece of scientific research with data that supports the conclusions. Experiments must have been conducted rigorously, with appropriate controls, replication, and sample sizes. The conclusions must be drawn appropriately based on the data presented.

Reviewer #1: Yes

Reviewer #2: Partly

**********

2. Has the statistical analysis been performed appropriately and rigorously?

Reviewer #1: Yes

Reviewer #2: I Don't Know

**********

3. Have the authors made all data underlying the findings in their manuscript fully available?

The PLOS Data policy requires authors to make all data underlying the findings described in their manuscript fully available without restriction, with rare exception (please refer to the Data Availability Statement in the manuscript PDF file). The data should be provided as part of the manuscript or its supporting information, or deposited to a public repository. For example, in addition to summary statistics, the data points behind means, medians and variance measures should be available. If there are restrictions on publicly sharing data—e.g. participant privacy or use of data from a third party—those must be specified.

Reviewer #1: Yes

Reviewer #2: No

**********

4. Is the manuscript presented in an intelligible fashion and written in standard English?

PLOS ONE does not copyedit accepted manuscripts, so the language in submitted articles must be clear, correct, and unambiguous. Any typographical or grammatical errors should be corrected at revision, so please note any specific errors here.

Reviewer #1: Yes

Reviewer #2: No

**********

5. Review Comments to the Author

Please use the space provided to explain your answers to the questions above. You may also include additional comments for the author, including concerns about dual publication, research ethics, or publication ethics. (Please upload your review as an attachment if it exceeds 20,000 characters)

Reviewer #1: Overall, the manuscript addresses an important public health question by examining the longitudinal relationship between waist-to-height ratio, physical activity, and cardiorespiratory fitness in a large cohort of Norwegian children.

The large sample size and longitudinal design of the HOPP study provide strong potential for meaningful insights into pediatric health risk indicators.

However, several methodological and reporting aspects require clarification, particularly regarding the statistical model structure, measurement procedures, and interpretation of regression results.

Strengthening these areas and moderating causal interpretations would significantly improve the scientific clarity and impact of the manuscript.

Abstract

1. The abstract contains an important methodological inconsistency: it states that WHtR was used as the independent variable, but then lists age, sex, MVPA, VO₂peak, and Andersen intermittent running test as dependent variables, which is conceptually unclear because age and sex are typically covariates or control variables rather than dependent outcomes. This needs clarification.

2. The results section of the abstract focuses on statistical significance but should better explain the practical meaning of the regression coefficients (e.g., how much fitness decreases with higher WHtR).

3. The conclusion should avoid implying a direct causal relationship between WHtR and lower physical activity or fitness, since the design primarily demonstrates associations within a longitudinal cohort.

Introduction

4. The introduction provides strong background on childhood obesity, physical inactivity, and cardiometabolic risk, but it places relatively little emphasis on the specific advantages of waist-to-height ratio compared with other adiposity indicators such as BMI or waist circumference alone. A clearer justification for selecting WHtR as the primary indicator would strengthen the rationale.

5. Several paragraphs broadly discuss physical activity and obesity in children but do not directly connect this information to the specific research objective of examining longitudinal relationships between WHtR, MVPA, and aerobic fitness. The section would benefit from a more focused narrative leading directly to the research question.

6. The final paragraph should more clearly state the specific hypotheses, particularly whether the authors expected WHtR to predict lower MVPA, lower VO₂peak, or both.

Methods

Study Design and Participants

7. The description of the Health Oriented Pedagogical Project (HOPP) cohort is informative, but the manuscript should more clearly explain the sampling framework and school recruitment procedures, including whether schools were randomly selected or volunteered to participate.

8. Because the study spans several years (2015–2020), it is important to clarify participant retention and attrition rates, including how many children were included in repeated measurements and how missing data were handled.

9. Additional detail about socioeconomic background or school characteristics would help contextualize the population and improve the interpretation of generalizability.

Measurements (WHtR, Physical Activity, Fitness)

10. The measurement procedures for waist circumference and height should provide clearer methodological detail (e.g., number of measurements taken, measurement reliability, and standardization procedures).

11. The manuscript should more clearly explain how physical activity (MVPA) was measured and processed, including accelerometer settings, epoch length, wear-time criteria, and cut-points used to classify MVPA.

12. The relationship between VO₂peak and the Andersen intermittent running test should be clarified, particularly whether VO₂peak was directly measured or estimated from the test performance.

Statistical Analysis

13. The manuscript states that linear mixed models (LMM) were used, which is appropriate for longitudinal data, but the description of the model structure requires clarification. Specifically, it should clearly indicate which variables were treated as fixed effects and which were included as random effects (e.g., participant or school level).

14. The analysis section should clarify whether time was included as a repeated factor in the model, given the longitudinal design of the HOPP dataset.

15. The manuscript should also explain how potential confounding variables such as age, sex, and growth-related changes were incorporated into the models.

Results

16. The results section clearly reports associations between WHtR and fitness-related variables, but the presentation would benefit from clearer interpretation of regression coefficients, explaining their practical meaning for child health and performance.

17. Given the large sample size (n ≈ 2297), the manuscript should place greater emphasis on effect size interpretation rather than relying primarily on statistical significance.

18. The relationship between WHtR, MVPA, and VO₂peak could be presented more clearly by emphasizing the direction and magnitude of associations rather than focusing primarily on p-values.

Discussion

19. The discussion appropriately highlights the relationship between central adiposity and reduced physical fitness, but the explanation of mechanisms could be expanded to include physiological and behavioral pathways linking adiposity to reduced physical activity and aerobic capacity.

20. The manuscript should more clearly compare the findings with previous research examining WHtR, BMI, and fitness in children, highlighting whether the current results confirm or extend existing evidence.

21. The limitations section should more explicitly acknowledge several important issues, including measurement limitations of accelerometry, potential residual confounding, and the observational nature of the data, which limits causal interpretation.

Practical Implications

22. The manuscript suggests that WHtR may be a useful screening tool for identifying children with lower physical activity and fitness; however, the practical implications should further explain how schools or health professionals could implement such screening in real-world settings.

23. The discussion could also address how school-based physical activity programs might target children with higher WHtR to improve both fitness and cardiometabolic health.

Conclusions

24. The conclusions should be slightly moderated to avoid implying that WHtR directly determines physical activity or fitness levels.

25. The section should emphasize that WHtR is associated with lower MVPA and lower cardiorespiratory fitness, highlighting its potential value as an early screening indicator for cardiometabolic risk in children.

Reviewer #2: This is an important and useful study examining an emerging measure of excess adiposity in detecting the risk of physical fitness, however, the authors submitted an embarrassing text. This reviewer provided comments with an interest in improving the study; otherwise, this is a straight rejection.

Major comments.

The article has no reference bibliography, the in-text citation has two types of referencing styles… This is unprofessional and sloppy. The authors should provide an explanation for such sloppiness.

Did the authors use ChatGPT to write the original draft of the article?

The authors should include the latest longitudinal evidence and reviews in the introductory rationale. Remove all references >10 years old. The field is rapidly evolving.

For example, rather than cite (Tremblay et al. 2011) in Line #98 on relations between movement behaviour and adiposity, a stronger long-term evidence exists in children, https://doi.org/10.1038/s41467-023-43316-w

A review on sedentary behaviour is also important to this work. https://doi.org/10.1152/physrev.00022.2022

Remove all references for adults from the text and focus on children and adolescents.

This statement has no meaning “Tests like 20 m SRT and Yo-Yo can be problematic from a pedagogical view.”… please delete. https://doi.org/10.1152/ajpheart.00570.2025

Focus on establishing the role of pediatric WHtR in detecting cardiometabolic risk, liver diseases, type 2 diabetes, and hypertension in the introductory rationale and adding these new references to both the introduction and discussion

https://doi.org/10.1038/s41366-025-01949-5

https://doi.org/10.1016/j.tjnut.2026.101426

https://doi.org/10.1093/obendo/wjaf002

https://doi.org/10.1210/jendso/bvaf079

“This study aims to test the WHtR’s ability to predict physical activity and fitness in children” is a weak justification. The authors should link the updated introduction to why this research question is important. One reason is the new approach to defining obesity, as emphasized in the new obesity guideline. Moreover, validating peadiatric WHtR cutoff as highlighted in the discussion is a valid research question.

Results:

Tables should be editable. The authors approach to copy paste photos or screenshots of table is very poor and suggests a lack of diligence.

What does the two repeated column of WHtR represent in Table 1?

Table 1 should present the baseline characteristics of the participants. It is shameful that there is no information.

Prepare a table showing the values of WHtR per quartile in this cohort.

Reanalyse your study using a pediatric validated cutoff which you have cited as reference #50.

What is the prevalence of high and excess fat in this population based on the Waist-to-height ratio cut points are <0.40 (low fat mass), 0.40 to < 0.50 (normal fat mass), 0.50 to < 0.53 (high fat mass), and ≥0.53 (excess fat mass) in males. Waist-to-height ratio cut points for females are <0.40 (low fat mass), 0.40 to < 0.51 (normal fat mass), 0.51 to < 0.54 (high fat mass), and ≥0.54 (excess fat mass)

The discussion should be entirely rewritten, not with chatGPT as it is too long and incoherent. Use the references suggested above to improve this manuscript.

is this a longitudinal study, what was the follow up duration. which measures are undertaken at baseline and follow-up.

Statistical analysis strategy must be well written for clarity and repeatability.

What does this mean? Automatisk oppdatering av henvisninger er deaktivert. For å se bibliografien, klikk Oppdater I Zotero fanen.

**********

6. PLOS authors have the option to publish the peer review history of their article (what does this mean?). If published, this will include your full peer review and any attached files.

If you choose “no”, your identity will remain anonymous but your review may still be made public.

**Do you want your identity to be public for this peer review?**   For information about this choice, including consent withdrawal, please see our Privacy Policy.

Reviewer #1: **Yes:**   Júlio Alejandro Henriques Castro da Costa

Reviewer #2: No

**********

[NOTE: If reviewer comments were submitted as an attachment file, they will be attached to this email and accessible via the submission site. Please log into your account, locate the manuscript record, and check for the action link "View Attachments". If this link does not appear, there are no attachment files.]

To ensure your figures meet our technical requirements, please review our figure guidelines: https://journals.plos.org/plosone/s/figures

You may also use PLOS’s free figure tool, NAAS, to help you prepare publication quality figures: https://journals.plos.org/plosone/s/figures#loc-tools-for-figure-preparation.

NAAS will assess whether your figures meet our technical requirements by comparing each figure against our figure specifications.

# Author comment: Author response to Decision Letter 1

**DOI:** 10.1371/journal.pone.0351792.r002

13 Apr 2026

Reviewer Comments and Responses

Reviewer #1

Comment #1

Overall, the manuscript addresses an important public health question by examining the longitudinal relationship between waist-to-height ratio, physical activity, and cardiorespiratory fitness in a large cohort of Norwegian children. The large sample size and longitudinal design of the HOPP study provide strong potential for meaningful insights into pediatric health risk indicators. However, several methodological and reporting aspects require clarification, particularly regarding the statistical model structure, measurement procedures, and interpretation of regression results. Strengthening these areas and moderating causal interpretations would significantly improve the scientific clarity and impact of the manuscript.

Response

Thank you for this balanced overall assessment. We agree that the manuscript would benefit from clearer reporting of the cohort design, measurement procedures, linear mixed-model structure, and interpretation of the regression coefficients. In the revised version, the methods and results sections are strengthened.

Comment #2

The abstract contains an important methodological inconsistency: it states that WHtR was used as the independent variable, but then lists age, sex, MVPA, VO₂peak, and Andersen intermittent running test as dependent variables, which is conceptually unclear because age and sex are typically covariates or control variables rather than dependent outcomes. This needs clarification.

Response

Thank you. The abstract is rewritten to distinguish clearly between exposure, outcomes, and covariates.

Comment #3

The results section of the abstract focuses on statistical significance but should better explain the practical meaning of the regression coefficients (e.g., how much fitness decreases with higher WHtR).

Response

We agree. The abstract is revised.

Comment #4

The conclusion should avoid implying a direct causal relationship between WHtR and lower physical activity or fitness, since the design primarily demonstrates associations within a longitudinal cohort.

Response

We agree. The Discussion and Conclusion are moderated.

Comment #5

The introduction provides strong background on childhood obesity, physical inactivity, and cardiometabolic risk, but it places relatively little emphasis on the specific advantages of waist-to-height ratio compared with other adiposity indicators such as BMI or waist circumference alone. A clearer justification for selecting WHtR as the primary indicator would strengthen the rationale.

Response

Thank you. We agree, and the rationale for selecting WHtR as the primary anthropometric indicator is strengthened.

Comment #6

Several paragraphs broadly discuss physical activity and obesity in children but do not directly connect this information to the specific research objective of examining longitudinal relationships between WHtR, MVPA, and aerobic fitness. The section would benefit from a more focused narrative leading directly to the research question.

Response

We agree. The introduction is tightened so that each paragraph leads directly to the study aim.

Comment #7

The final paragraph should more clearly state the specific hypotheses, particularly whether the authors expected WHtR to predict lower MVPA, lower VO₂peak, or both.

Response

We agree. The final paragraph of the introduction is revised to state the specific hypotheses explicitly.

Comment #8

The description of the Health Oriented Pedagogical Project (HOPP) cohort is informative, but the manuscript should more clearly explain the sampling framework and school recruitment procedures, including whether schools were randomly selected or volunteered to participate.

Response

Thank you. We agree that the recruitment framework should be described directly in this manuscript rather than only by citation to earlier HOPP papers. We clarify that the cohort was school-based and non-random, that all elementary schools in Horten municipality participated in the intervention arm, and that two comparison schools from the greater Oslo area served as controls.

Comment #9

Because the study spans several years (2015–2020), it is important to clarify participant retention and attrition rates, including how many children were included in repeated measurements and how missing data were handled.

Response

We agree. The revised manuscript now includes a table showing the number of participants per year.

Comment #10

Additional detail about socioeconomic background or school characteristics would help contextualize the population and improve the interpretation of generalizability.

Response

We agree. A short contextual description of the participating schools and socioeconomic background is added.

Comment #11

The measurement procedures for waist circumference and height should provide clearer methodological detail (e.g., number of measurements taken, measurement reliability, and standardization procedures).

Response

We agree. The anthropometric methods are expanded.

Comment #12

The manuscript should more clearly explain how physical activity (MVPA) was measured and processed, including accelerometer settings, epoch length, wear-time criteria, and cut-points used to classify MVPA.

Response

We believe this is explained in the Methods section under “Physical activity.”

Comment #13

The relationship between VO₂peak and the Andersen intermittent running test should be clarified, particularly whether VO₂peak was directly measured or estimated from the test performance.

Response

We believe this is already stated in the text. The description of the VO₂peak test is provided, as well as the Andersen test. To be clear, as written in the text, VO₂peak was not estimated from the Andersen test.

Comment #14

The manuscript states that linear mixed models (LMM) were used, which is appropriate for longitudinal data, but the description of the model structure requires clarification. Specifically, it should clearly indicate which variables were treated as fixed effects and which were included as random effects (e.g., participant or school level).

Response

We agree. The mixed-model specification is rewritten for clarity. The revised text states that repeated observations were nested within participants, participant ID was included as a random intercept, and WHtR, age, and sex were included as fixed effects. Because the number of schools was small, school is best addressed as a sensitivity covariate or fixed effect rather than as a higher-level random effect.

Comment #15

The analysis section should clarify whether time was included as a repeated factor in the model, given the longitudinal design of the HOPP dataset.

Response

We agree, and the text is changed to better describe the analyses.

Comment #16

The manuscript should also explain how potential confounding variables such as age, sex, and growth-related changes were incorporated into the models.

Response

This is now explained.

Comment #17

The results section clearly reports associations between WHtR and fitness-related variables, but the presentation would benefit from clearer interpretation of regression coefficients, explaining their practical meaning for child health and performance.

Response

We agree, and relevant changes have been made in the Results section.

Comment #18

Given the large sample size (n ≈ 2297), the manuscript should place greater emphasis on effect size interpretation rather than relying primarily on statistical significance.

Response

We agree. The text has now been changed to better accommodate effect size interpretation.

Comment #19

The relationship between WHtR, MVPA, and VO₂peak could be presented more clearly by emphasizing the direction and magnitude of associations rather than focusing primarily on p-values.

Response

We agree and revised the Results narrative accordingly.

Comment #20

The discussion appropriately highlights the relationship between central adiposity and reduced physical fitness, but the explanation of mechanisms could be expanded to include physiological and behavioral pathways linking adiposity to reduced physical activity and aerobic capacity.

Response

Thank you. We agree and expand the discussion of plausible mechanisms.

Comment #21

The manuscript should more clearly compare the findings with previous research examining WHtR, BMI, and fitness in children, highlighting whether the current results confirm or extend existing evidence.

Response

We agree. The revised Discussion compares the present findings more systematically with previous pediatric studies on WHtR, BMI, and fitness.

Comment #22

The limitations section should more explicitly acknowledge several important issues, including measurement limitations of accelerometry, potential residual confounding, and the observational nature of the data, which limits causal interpretation.

Response

We agree. The limitations section is now expanded substantially.

Comment #23

The manuscript suggests that WHtR may be a useful screening tool for identifying children with lower physical activity and fitness; however, the practical implications should further explain how schools or health professionals could implement such screening in real-world settings.

Response

We agree. The practical implications section is now made more concrete.

Comment #24

The discussion could also address how school-based physical activity programs might target children with higher WHtR to improve both fitness and cardiometabolic health.

Response

We agree. A short paragraph on how school-based interventions may respond to these findings without stigmatizing children is added to the Discussion.

Comment #25

The conclusions should be slightly moderated to avoid implying that WHtR directly determines physical activity or fitness levels.

Response

We agree, and the Conclusion is now moderated.

Comment #26

The section should emphasize that WHtR is associated with lower MVPA and lower cardiorespiratory fitness, highlighting its potential value as an early screening indicator for cardiometabolic risk in children.

Response

This is now included in the Conclusion.

Reviewer #2

Comment #1

This is an important and useful study examining an emerging measure of excess adiposity in detecting the risk of physical fitness; however, the authors submitted an embarrassing text. This reviewer provided comments with an interest in improving the study; otherwise, this is a straight rejection.

Response

We thank the reviewer for recognizing the importance of the study and for providing detailed comments intended to improve the manuscript. We acknowledge that the submitted version contained major formatting and presentation problems, particularly in the reference list and tables, and we regret that these technical issues detracted from the scientific content. The revised version is substantially cleaned, reformatted, and rewritten for clarity.

Comment #2

The article has no reference bibliography, the in-text citation has two types of referencing styles. This is unprofessional and sloppy. The authors should provide an explanation for such sloppiness.

Response

We apologize for this formatting error. The problem was not intentional and appears to have resulted from a Zotero/Word field-update issue in the submitted version, where automatic reference updating was disabled before export. This prevented the bibliography and citation fields from refreshing correctly. The reference list and citation style have now been corrected and unified throughout the manuscript.

Comment #3

Did the authors use ChatGPT to write the original draft of the article?

Response

No. The manuscript was not written by generative AI. As already disclosed in the manuscript’s AI statement, ChatGPT was used only for limited English-language correction, after which the authors reviewed and edited the text and retained full responsibility for the content, interpretation, and final wording.

Comment #4

The authors should include the latest longitudinal evidence and reviews in the introductory rationale. Remove all references >10 years old. The field is rapidly evolving.

Response

We agree in part. The Introduction and Discussion are updated with more recent longitudinal evidence and recent reviews, particularly regarding movement behavior, pediatric adiposity, and WHtR-based risk classification. However, we do not believe that all references older than 10 years should automatically be removed, because some older sources are foundational method papers, such as the Andersen test, the 20 m shuttle run, and original WHtR rationale papers.

Comment #5

For example, rather than cite (Tremblay et al. 2011) in Line #98 on relations between movement behaviour and adiposity, a stronger long-term evidence exists in children, https://doi.org/10.1038/s41467-023-43316-w

Response

We agree and have supplemented the older citation with the more recent longitudinal pediatric evidence.

Comment #6

A review on sedentary behaviour is also important to this work. https://doi.org/10.1152/physrev.00022.2022

Response

We agree, and Pinto et al. is added, especially to frame why sedentary time may not be equivalent to low MVPA and why physiology- and posture-specific mechanisms matter.

Comment #7

Remove all references for adults from the text and focus on children and adolescents.

Response

We agree in principle. The revised manuscript focuses more consistently on pediatric evidence. Two methodological or conceptual references are retained.

Comment #8

This statement has no meaning: “Tests like 20 m SRT and Yo-Yo can be problematic from a pedagogical view.” Please delete. https://doi.org/10.1152/ajpheart.00570.2025

Response

We do not agree with this point in principle. Tests that exclude children during the assessment because they fail to maintain the required pace may be problematic in pediatric populations, as they can stigmatize children with lower physical capacity. One advantage of the Andersen test is that all children are able to complete the test. However, we agree that this point is not essential to the scientific argument of the manuscript, and the statement is therefore deleted.

Comment #9

Focus on establishing the role of pediatric WHtR in detecting cardiometabolic risk, liver diseases, type 2 diabetes, and hypertension in the introductory rationale, and add these new references to both the Introduction and Discussion: https://doi.org/10.1038/s41366-025-01949-5 https://doi.org/10.1016/j.tjnut.2026.101426 https://doi.org/10.1093/obendo/wjaf002 https://doi.org/10.1210/jendso/bvaf079

Response

Thank you. We agree that the rationale should be strengthened by linking WHtR not only to adiposity, but also to clinically relevant pediatric outcomes. The Introduction and Discussion are revised accordingly.

Comment #10

“This study aims to test the WHtR’s ability to predict physical activity and fitness in children” is a weak justification. The authors should link the updated introduction to why this research question is important. One reason is the new approach to defining obesity, as emphasized in the new obesity guideline. Moreover, validating pediatric WHtR cutoff, as highlighted in the discussion, is a valid research question.

Response

We agree, and the wording is now changed.

Comment #11

Tables should be editable. The authors’ approach of copy-pasting photos or screenshots of tables is very poor and suggests a lack of diligence.

Response

We agree and apologize. All tables are now editable.

Comment #12

What does the two repeated column of WHtR represent in Table 1?

Response

This appears to be a table-formatting or labeling error and is now corrected.

Comment #13

Table 1 should present the baseline characteristics of the participants. It is shameful that there is no information.

Response

We agree. A table presenting baseline participant characteristics is added (now Table 2).

Comment #14

Prepare a table showing the values of WHtR per quartile in this cohort.

Response

We agree, but rather than a table we included the results in the figures. All figures now also include all values per WHtR quartile.

Comment

Attachment Submitted filename: Reviewer comments and responses.docx

# Peer review: Decision Letter 1

**DOI:** 10.1371/journal.pone.0351792.r003

**Authors:** Timoteo Salvador Lucas Daca

28 Apr 2026

PONE-D-26-07428R1

Waist-to-height ratio identifies children with lower physical activity and reduced cardiorespiratory fitness: longitudinal evidence from Norwegian primary schools – the Health Oriented Pedagogical Project (HOPP).

PLOS One

Dear Dr. Fredriksen,

Thank you for submitting your manuscript to PLOS ONE. After careful consideration, we feel that it has merit but does not fully meet PLOS ONE’s publication criteria as it currently stands. Therefore, we invite you to submit a revised version of the manuscript that addresses the points raised during the review process.

There are a few minor and a major comment pointed out by Reviewer 2 that need to be attended to, including spelling errors, same changes, and improving the discussion section!

Please submit your revised manuscript by Jun 12 2026 11:59PM.  If you will need more time than this to complete your revisions, please reply to this message or contact the journal office at plosone@plos.org. When you're ready to submit your revision, log on to https://www.editorialmanager.com/pone/ and select the 'Submissions Needing Revision' folder to locate your manuscript file.

Please include the following items when submitting your revised manuscript:

- A letter that responds to each point raised by the academic editor and reviewer(s). You should upload this letter as a separate file labeled 'Response to Reviewers'.
- A marked-up copy of your manuscript that highlights changes made to the original version. You should upload this as a separate file labeled 'Revised Manuscript with Track Changes'.
- An unmarked version of your revised paper without tracked changes. You should upload this as a separate file labeled 'Manuscript'.

If you would like to make changes to your financial disclosure, please include your updated statement in your cover letter. Guidelines for resubmitting your figure files are available below the reviewer comments at the end of this letter.

If applicable, we recommend that you deposit your laboratory protocols in protocols.io to enhance the reproducibility of your results. Protocols.io assigns your protocol its own identifier (DOI) so that it can be cited independently in the future. For instructions see: https://journals.plos.org/plosone/s/submission-guidelines#loc-laboratory-protocols. Additionally, PLOS ONE offers an option for publishing peer-reviewed Lab Protocol articles, which describe protocols hosted on protocols.io. Read more information on sharing protocols at https://plos.org/protocols?utm_medium=editorial-email&utm_source=authorletters&utm_campaign=protocols.

As the corresponding author, your ORCID iD is verified in the submission system and will appear in the published article. PLOS supports the use of ORCID, and we encourage all coauthors to register for an ORCID iD and use it as well. Please encourage your coauthors to verify their ORCID iD within the submission system before final acceptance, as unverified ORCID iDs will not appear in the published article. *Only*   the individual author can complete the verification step; PLOS staff *cannot*   verify ORCID iDs on behalf of authors.

We look forward to receiving your revised manuscript.

Kind regards,

Timoteo Salvador Lucas Daca, Ph.D

Academic Editor

PLOS One

Journal Requirements:

If the reviewer comments include a recommendation to cite specific previously published works, please review and evaluate these publications to determine whether they are relevant and should be cited. There is no requirement to cite these works unless the editor has indicated otherwise.

[Note: HTML markup is below. Please do not edit.]

Reviewers' comments:

Reviewer's Responses to Questions

**Comments to the Author**

1. If the authors have adequately addressed your comments raised in a previous round of review and you feel that this manuscript is now acceptable for publication, you may indicate that here to bypass the “Comments to the Author” section, enter your conflict of interest statement in the “Confidential to Editor” section, and submit your "Accept" recommendation.

Reviewer #1: All comments have been addressed

Reviewer #2: (No Response)

**********

2. Is the manuscript technically sound, and do the data support the conclusions?

The manuscript must describe a technically sound piece of scientific research with data that supports the conclusions. Experiments must have been conducted rigorously, with appropriate controls, replication, and sample sizes. The conclusions must be drawn appropriately based on the data presented.

Reviewer #1: Yes

Reviewer #2: (No Response)

**********

3. Has the statistical analysis been performed appropriately and rigorously?

Reviewer #1: Yes

Reviewer #2: (No Response)

**********

4. Have the authors made all data underlying the findings in their manuscript fully available?

The PLOS Data policy requires authors to make all data underlying the findings described in their manuscript fully available without restriction, with rare exception (please refer to the Data Availability Statement in the manuscript PDF file). The data should be provided as part of the manuscript or its supporting information, or deposited to a public repository. For example, in addition to summary statistics, the data points behind means, medians and variance measures should be available. If there are restrictions on publicly sharing data—e.g. participant privacy or use of data from a third party—those must be specified.

Reviewer #1: Yes

Reviewer #2: (No Response)

**********

5. Is the manuscript presented in an intelligible fashion and written in standard English?

PLOS ONE does not copyedit accepted manuscripts, so the language in submitted articles must be clear, correct, and unambiguous. Any typographical or grammatical errors should be corrected at revision, so please note any specific errors here.

Reviewer #1: Yes

Reviewer #2: (No Response)

**********

6. Review Comments to the Author

Please use the space provided to explain your answers to the questions above. You may also include additional comments for the author, including concerns about dual publication, research ethics, or publication ethics. (Please upload your review as an attachment if it exceeds 20,000 characters)

Reviewer #1: I am satisfied with the current version of the manuscript.

The authors have done a thorough job in revising the paper and have adequately addressed all the questions that were raised.

Reviewer #2: Thank you for the significant upgrade of the manuscript, which makes for a good read. A few minor and a major comment are listed herewith.

Line 78… spelling error ”therefor”

Line 88… change “closely tied to” two modifiable factors “of” future cardiometabolic health

Line 129… It appears that something is missing in the text “only children born 200 (the youngest)”

Line 182… typo error “HOPP’s primarily objective”… should be “primary”.

Line 228… typo error "from HOPP provid an overview”…. should be “provide”.

In the discussion, the authors should emphasise the significance of WHtR which was confirmed in this study regarding how WHtR overcame a major limitation of BMI variability with age and sex. The authors noted in the result section that “WHtR remains stable over time for both sexes” See the following references… https://doi.org/10.1038/s41366-025-01949-5

https://doi.org/10.1038/s41390-024-03112-8

Table 2 should include a column of mean age and SD of the boys and girls per study year and SES.

Also mean (SD) for MVPA, VO2 peak and Sedentary time must be included in Table 2 per year of follow up.

There are two symbols in the result Line 243-247 that should be defined at first use (β,d)

This point should be included in the abstract “Each 0.1 unit increase in WHtR was linked to a -0.62 min/day reduction in average MVPA”

The title of Table 3 reads more like a footnote, kindly revise.

The major concern is that representing WHtR in quartile is not clinically relevant. The topmost quartile equals 75th percentile. This lumps together normal WHtR fat mass, high fat mass and excess fat mass in the same category. Therefore, creating room for misclassifications. It is also impossible to state the role of excess adiposity on MVPA clearly.

Importantly, the authors response to the previous comment is not satisfactory “What is the prevalence of high and excess fat in this population based on the waist-to-height ratio cutpoints: <0.40 (low fat mass), 0.40 to <0.50 (normal fat mass), 0.50 to <0.53 (high fat mass), and ≥0.53 (excess fat mass) in males? Waist-to-height ratio cut points for females are <0.40 (low fat mass), 0.40 to <0.51 (normal fat mass), 0.51 to <0.54 (high fat mass), and ≥0.54 (excess fat mass).

This is the clinical relevance of this new study. These WHtR cutpoints represent 85th percentile to <95th percentile for high fat mass and 95th percentile and above as excess fat mass.

Rather than use quartile, this validated clinical cutpoints provide detailed information that could make this study a reference in future clinical guideline update. Kindly re-analyse the data based in the provide cutpoint.

This statement was repeated several times in the discussion, kindly delete all redundant repetitions “these results support WHtR as a simple marker that may help identify children at risk”

The nul association between sedentary behaviour and WHtR may be due to the quartile category approach which does not clearly delineate between real excess adiposity. Hence the clinical relevant cutpoints should be used to reexamine the associations of sedentary time and WHtR.

**********

7. PLOS authors have the option to publish the peer review history of their article (what does this mean?). If published, this will include your full peer review and any attached files.

If you choose “no”, your identity will remain anonymous but your review may still be made public.

**Do you want your identity to be public for this peer review?**   For information about this choice, including consent withdrawal, please see our Privacy Policy.

Reviewer #1: **Yes:**   Júlio Alejandro Henriques Castro da Costa

Reviewer #2: No

**********

[NOTE: If reviewer comments were submitted as an attachment file, they will be attached to this email and accessible via the submission site. Please log into your account, locate the manuscript record, and check for the action link "View Attachments". If this link does not appear, there are no attachment files.]

To ensure your figures meet our technical requirements, please review our figure guidelines: https://journals.plos.org/plosone/s/figures

You may also use PLOS’s free figure tool, NAAS, to help you prepare publication quality figures: https://journals.plos.org/plosone/s/figures#loc-tools-for-figure-preparation.

NAAS will assess whether your figures meet our technical requirements by comparing each figure against our figure specifications.

# Author comment: Author response to Decision Letter 2

**DOI:** 10.1371/journal.pone.0351792.r004

3 May 2026

Response to reviewers

Title: Waist-to-height ratio identifies children with lower physical activity and reduced cardiorespiratory fitness: longitudinal evidence from Norwegian primary schools – the Health Oriented Pedagogical Project (HOPP)

Reviewer #1

We thank Reviewer #1 for the careful evaluation of the revised manuscript and for the positive assessment.

Response: We are grateful for this encouraging assessment and thank the reviewer for recognizing the revisions made to improve the manuscript.

Reviewer #2

We thank Reviewer #2 for the positive overall assessment of the revised manuscript and for the remaining helpful comments. Each point is addressed below.

Comment 1

Comment: Line 78… spelling error “therefor”

Response: Thank you. This has been corrected to “therefore.”

Comment 2

Comment: Line 88… change “closely tied to” two modifiable factors “of” future cardiometabolic health

Response: Thank you. The sentence has been revised accordingly for clarity and grammatical correctness.

Comment 3

Comment: Line 129… It appears that something is missing in the text “only children born 200 (the youngest)”

Response: Thank you. This error has been corrected in the revised manuscript.

Comment 4

Comment: Line 182… typo error “HOPP’s primarily objective”… should be “primary”.

Response: Thank you. This has been corrected.

Comment 5

Comment: Line 228… typo error “from HOPP provid an overview”…. should be “provide”.

Response: Thank you. This has been corrected.

Comment 6

Comment: In the discussion, the authors should emphasise the significance of WHtR which was confirmed in this study regarding how WHtR overcame a major limitation of BMI variability with age and sex. The authors noted in the result section that “WHtR remains stable over time for both sexes” See the following references.

Response: We agree that one of the practical strengths of WHtR is that, unlike BMI, it is less dependent on age- and sex-specific interpretation across childhood. We have strengthened the discussion to emphasize this point more clearly and have incorporated the suggested references where relevant. This addition helps position WHtR more explicitly as a clinically useful marker that may overcome an important limitation of BMI in pediatric screening.

Comment 7

Comment: Table 2 should include a column of mean age and SD of the boys and girls per study year and SES. Also mean (SD) for MVPA, VO2 peak and Sedentary time must be included in Table 2 per year of follow up.

Response: We agree that clearer descriptive information improves interpretability. We have revised the descriptive presentation accordingly in the table. The SES was registered in 2015, and the distribution is mentioned in the method section.

Comment 8

Comment: There are two symbols in the result Line 243–247 that should be defined at first use (β, d).

Response: Thank you. We have now defined β and d in the Method section.

Comment 9

Comment: This point should be included in the abstract “Each 0.1 unit increase in WHtR was linked to a -0.62 min/day reduction in average MVPA”

Response: We agree that the abstract should reflect the main quantitative finding more clearly. We have revised the abstract to report the WHtR–MVPA association in a clearer and more interpretable form.

Comment 10

Comment: The title of Table 3 reads more like a footnote, kindly revise.

Response: Thank you. The title of Table 3 has been revised to make it shorter and more consistent with standard table formatting.

Comment 11

Comment: The major concern is that representing WHtR in quartile is not clinically relevant. The topmost quartile equals 75th percentile. This lumps together normal WHtR fat mass, high fat mass and excess fat mass in the same category. Therefore, creating room for misclassifications. It is also impossible to state the role of excess adiposity on MVPA clearly.

Importantly, the authors response to the previous comment is not satisfactory “What is the prevalence of high and excess fat in this population based on the waist-to-height ratio cutpoints: <0.40 (low fat mass), 0.40 to <0.50 (normal fat mass), 0.50 to <0.53 (high fat mass), and ≥0.53 (excess fat mass) in males? Waist-to-height ratio cut points for females are <0.40 (low fat mass), 0.40 to <0.51 (normal fat mass), 0.51 to <0.54 (high fat mass), and ≥0.54 (excess fat mass).

This is the clinical relevance of this new study. These WHtR cutpoints represent 85th percentile to <95th percentile for high fat mass and 95th percentile and above as excess fat mass. Rather than use quartile, this validated clinical cutpoints provide detailed information that could make this study a reference in future clinical guideline update. Kindly re-analyse the data based in the provide cutpoint.

Response: We thank the reviewer for this thoughtful comment and for highlighting the potential clinical relevance of recently proposed WHtR cut-points. We agree that such cut-points may be useful when the purpose is to classify children into adiposity categories or to estimate the prevalence of high and excess fat mass. However, that is not the primary aim of the present manuscript.

Our study was designed to examine associations between WHtR and physical activity and fitness, not to compare predefined adiposity classes. In this context, WHtR was analysed primarily as a continuous exposure variable in the linear mixed models, which we consider the most appropriate approach for addressing the study question. The quartile presentation was included only as a descriptive and visual illustration of the graded decline in physical activity and fitness across the WHtR distribution. It was not intended as a clinical classification system.

We therefore respectfully disagree that the use of quartiles constitutes “misclassification” in the context of this manuscript. Quartiles were not used to define clinical fat-mass categories, but to display the pattern of association across the observed WHtR range. The main message is the graded relationship between increasing WHtR and lower MVPA and fitness, rather than the contrast between discrete adiposity categories.

We also examined the reviewer’s suggested WHtR cut-points in our dataset. These additional analyses did not provide materially different information from the analyses already presented. Rather, they confirmed the same overall pattern: higher WHtR was associated with higher adiposity and less favourable body-composition measures, which is fully consistent with the findings from the continuous models and quartile-based descriptive presentation. In our view, reframing the manuscript around multiple WHtR cut-points would shift the emphasis from association to classification without adding substantial interpretive value to the core message of the study.

We further note that, from a public health perspective, the WHtR threshold of 0.5 remains the most widely recognized and easily interpretable message. While more granular cut-points may be of theoretical and clinical interest, especially in body-fat classification research, their practical utility in public communication is less straightforward. For this reason, we believe that the present analytical approach better serves the aims of the manuscript.

Comment 12

Comment: This statement was repeated several times in the discussion, kindly delete all redundant repetitions “these results support WHtR as a simple marker that may help identify children at risk”

Response: We agree that this point was stated too often in the previous version. Redundant repetitions have been removed.

Comment 13

Comment: The null association between sedentary behaviour and WHtR may be due to the quartile category approach …

Response: We appreciate this point. However, the null finding for sedentary behavior was not based on the quartile figures, but on the linear mixed models in which WHtR was analysed as a continuous variable. The quartile presentation was descriptive only. We have clarified this more explicitly in the revised manuscript to avoid the impression that the null association was an artifact of categorization.

Attachment Submitted filename: Response 2 referees.docx

# Peer review: Decision Letter 2

**DOI:** 10.1371/journal.pone.0351792.r005

**Authors:** Timoteo Salvador Lucas Daca

1 Jun 2026

Waist-to-height ratio identifies children with lower physical activity and reduced cardiorespiratory fitness: longitudinal evidence from Norwegian primary schools – the Health Oriented Pedagogical Project (HOPP).

PONE-D-26-07428R2

Dear Dr. Fredriksen,

We’re pleased to inform you that your manuscript has been judged scientifically suitable for publication and will be formally accepted for publication once it meets all outstanding technical requirements. Within one week, you’ll receive an e-mail detailing the required amendments. When these have been addressed, you’ll receive a formal acceptance letter and your manuscript will be scheduled for publication.  An invoice will be generated when your article is formally accepted. Please note, if your institution has a publishing partnership with PLOS and your article meets the relevant criteria, all or part of your publication costs will be covered. Please make sure your user information is up-to-date by logging into Editorial Manager at Editorial Manager® and clicking the ‘Update My Information' link at the top of the page. For questions related to billing, please contact billing support.

If your institution or institutions have a press office, please notify them about your upcoming paper to help maximize its impact. If they’ll be preparing press materials, please inform our press team as soon as possible -- no later than 48 hours after receiving the formal acceptance. Your manuscript will remain under strict press embargo until 2 pm Eastern Time on the date of publication. For more information, please contact onepress@plos.org.

Kind regards,

Timoteo Salvador Lucas Daca, Ph.D

Academic Editor

PLOS One

# Editor report: Acceptance letter

**DOI:** 10.1371/journal.pone.0351792.r006

**Authors:** Timoteo Salvador Lucas Daca

PONE-D-26-07428R2

PLOS One

Dear Dr. Fredriksen,

I'm pleased to inform you that your manuscript has been deemed suitable for publication in PLOS One. Congratulations! Your manuscript is now being handed over to our production team.

At this stage, our production department will prepare your paper for publication. This includes ensuring the following:

* All references, tables, and figures are properly cited

* All relevant supporting information is included in the manuscript submission,

* There are no issues that prevent the paper from being properly typeset

You will receive further instructions from the production team, including instructions on how to review your proof when it is ready. Please keep in mind that we are working through a large volume of accepted articles, so please give us a few days to review your paper and let you know the next and final steps.

Lastly, if your institution or institutions have a press office, please let them know about your upcoming paper now to help maximize its impact. If they'll be preparing press materials, please inform our press team within the next 48 hours. Your manuscript will remain under strict press embargo until 2 pm Eastern Time on the date of publication. For more information, please contact onepress@plos.org.

You will receive an invoice from PLOS for your publication fee after your manuscript has reached the completed accept phase. If you receive an email requesting payment before acceptance or for any other service, this may be a phishing scheme. Learn how to identify phishing emails and protect your accounts at https://explore.plos.org/phishing.

If we can help with anything else, please email us at customercare@plos.org.

Thank you for submitting your work to PLOS ONE and supporting open access.

Kind regards,

PLOS ONE Editorial Office Staff

on behalf of

Dr. Timoteo Salvador Lucas Daca

Academic Editor

PLOS One
